# Identification of prosthetic joint infection by *Candida* using metagenomic shotgun sequencing

**DOI:** 10.1099/acmi.0.000425

**Published:** 2023-10-26

**Authors:** Liqiong Lin, Kaiwei Shen, Lili Xiao, Yu lin, Eryou Feng, Yiyuan Zhang

**Affiliations:** ^1^​ Department of Orthopedics, Fuzhou Second Hospital, Fuzhou, PR China; ^2^​ Fujian Provincial Clinical Medical Research Center for First Aid and Rehabilitation in Orthopaedic Trauma, Fuzhou, PR China; ^3^​ Institute for Biochemistry and Molecularbiology, ZBMZ, Faculty of Medicine, Albert-Ludwigs-Universität Freiburg, Freiburg, Germany

**Keywords:** *Candida*, metagenomic shotgun sequencing, prosthetic joint infection

## Abstract

**Background.:**

Periprosthetic joint infection (PJI) is a serious complication after total knee arthroplasty. Fungal infections are prone to biofilm formation, which makes it hard to diagnose and clarify the pathogenic species.

**Case Presentation.:**

This case study provides evidence of a novel PJI pathogen that is otherwise difficult to detect using conventional methods. A patient was reviewed with persistent postoperative pain, swelling and eventually drainage around the left knee after undergoing a bilateral total knee arthroplasty 2 years previously for progressive osteoarthritis. By using metagenomic shotgun sequencing to analyse both bacterial and fungal agent sequences, we were able to identify fungal strains of *Candida tropicalis*, a rarely reported and difficult-to-culture PJI pathogen.

**Conclusion.:**

Metagenomic shotgun sequencing enables the detection of difficult-to-detect pathogens and the formulation of treatment recommendations for fungal infections with low positive rates based on gene content analysis.

## Data Summary

The GenBank accession numbers for the metagenomic shotgun sequencing raw databases are available at the NCBI database (https://www.ncbi.nlm.nih.gov). The reference genomes for human host sequences are available at BioProject (https://www.ncbi.nlm.nih.gov/bioproject/?term=PRJNA976639).

## Introduction

Periprosthetic joint infection (PJI) occurs in 1–2 % of primary joint replacements, with a 5–40 % occurrence rate in revision cases [1]. It is currently the major cause of failure following total knee arthroplasty [1]. A wide variety of pathogens has been found to cause PJI, with the majority caused by Gram-positive bacteria, in particular *Staphylococcus* spp. [2]. Despite the fact that fungal infections are rare and occur in approximately 1 % of all PJI, they are becoming increasingly prevalent [3]. *Candida albicans* is the most frequent cause of fungal PJI. *Candida* species are the primary causative agents in at least 80 % of these cases [4]. We present a report on patients with *Candida tropicalis* PJIs, determined by metagenomic shotgun sequencing to examine the pathogenic gene sequence. *C. tropicalis* is a rarely reported and difficult-to-culture PJI pathogen.

## Case report

A 68-year-old woman was referred to our Department of Orthopaedic Surgery Group for evaluation of a chronic left knee PJI. She had a significant medical history, including diabetes that had been diagnosed 9 years earlier and was in remission after treatment with insulin and coronary heart disease that had been diagnosed 8 years earlier and was being treated with percutaneous coronary intervention (PCI) and clopidogrel 50 mg once daily over a long period, along with lercanidipine 10 mg per day and losartan 50 mg per day. Based on data from her previous hospital treatment, she had undergone bilateral total knee arthroplasty (TKA) at an outside institution 2 years prior due to osteoarthritis in both knees. After the TKA, she developed pain, swelling and eventually drainage from a sinus tract around the left knee. Cultures of synovial fluid aspirates revealed fungal infections. (The original culture report was not available, and specific information could not be retrieved as it has been simply described as a fungal infection in her previous treatment records.) Over the following 2 years, she was prescribed multiple oral antifungal drugs and joint cavity injection therapy, but there was little improvement. As a result, she was referred to us for further assessment and treatment.

At the time of referral, the patient reported ongoing knee pain, with no fevers or chills. A physical examination indicated slight crusting over the anterior of the left patella. The results of the laboratory evaluation were considerable, with an erythrocyte sedimentation rate (ESR) of 40 mm h^−1^ (normal range, 0–20 mm h^−1^) and C-reactive protein (CRP) level of 3.22 mg l^−1^ (normal, 0–8.0 mg l^−1^). Synovial fluid analysis exhibited 6977 cells mm^−3^ (normal, < 3000 cells mm^−3^) with 57 % neutrophils (normal, <70 %), aerobic and anaerobic bacterial cultures were negative, and *C. tropicalis* was found in the fungal culture. At the bone–cement interface of the tibial component, a radiolucent line was seen in radiographs, with reactive sclerotic changes that might suggest septic loosening. The inflammatory arthritis treatment was discontinued and she was kept additional antibiotics in anticipation of surgery. The patient had her TKA resected 5 months later as part of a two-stage exchange arthroplasty procedure. Purulent material and necrotic tissue were observed during surgery. Pathology revealed acute and chronic inflammation.

**Fig. 1.**
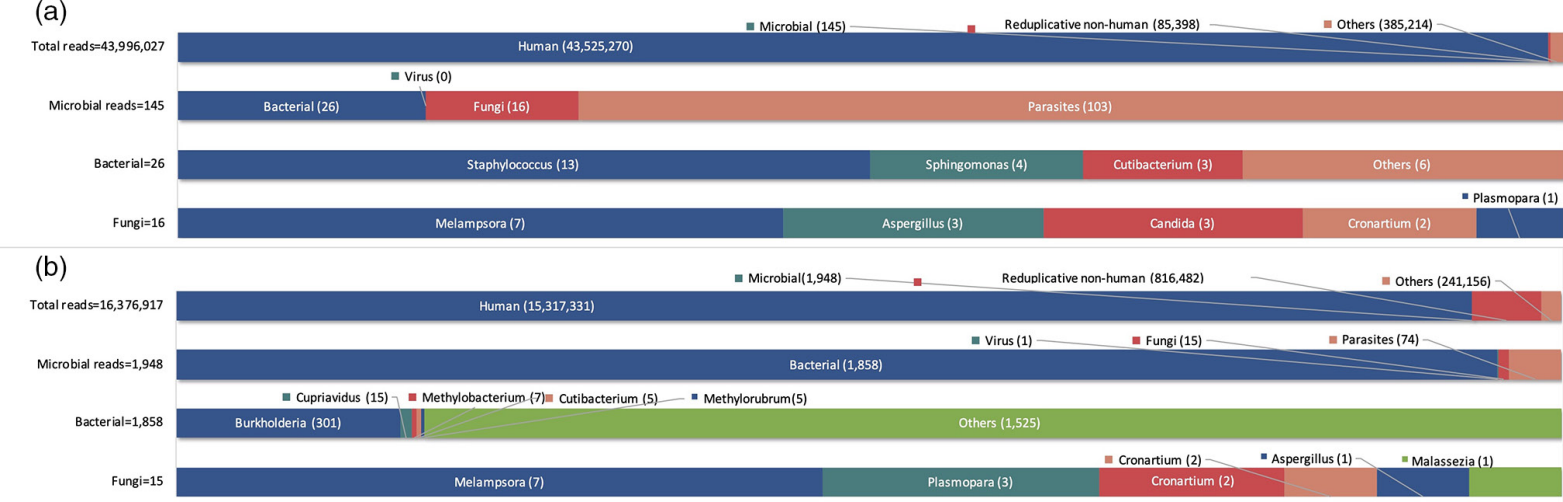
Taxonomic classification of reads by bioinformatic analysis of sequenced data from intraoperative synovial fluid from 14 April 2020 (a) and intraoperative synovial fluid from 15 July 2020 (b).

Knee implants were removed from lesion debridement intraoperatively through the use of a femoral knee prosthesis with a tibial rotating platform liner and no tibial prosthesis with bone cement, as part of a planned two-stage exchange arthroplasty. Purulent material, as well as necrotic tissue, were found intraoperatively. Three specimens of periprosthetic tissue were cultured for 2 weeks. Both the aerobic and anaerobic bacterial cultures were negative, whereas *Candida* was detected in the fungal cultures. One of these samples was maintained at −80 °C for the metagenomic shotgun sequencing (mNGS) test. Lyticase lysoase (RT410-TA, TIANGEN BIOTECH, Beijing, PR China) was employed to break down the enzyme wall. Following the second step of DNA extraction recommended by the TIANamp Micro DNA kit (DP316, TIANGEN BIOTECH, Beijing, PR China), the sample was sequenced utilizing the BGISEQ-50/MGISEQ-2000 system [5]. Furthermore, in line with the use of the BWA alignment, which can be found at http://bio-bwa.sourceforge.net [6], the remaining data were matched with the Huada PMDB pathogen database after low-complexity reads had been eliminated. A total of 43 996 027 reads were obtained from periprosthetic tissue, and *C. tropicalis* was detected, with 3 reads collected from this organism (2.08 % of microbial reads, 18.75 % of fungal reads). The other micro-organisms detected by mNGS are listed in Table 1 and Fig. 1, although they were not thought to be responsible for the PJI. As a result, we regarded *C. tropicalis* as the definite causative pathogen.

**Table 1.** The metagenomic shotgun sequencing report from 14 April 2020

Type	Genus	No. of reads	Species	Coverage rate (%)	Depth	No. of reads
Bacteria	* Staphylococcus *	13	*Hominis*	1.39	1	7
	* Sphingomonas *	4	*Echinoides*	0.90	1	4
	* Ralstonia *	41	*Pickettii*	2.82	1	35
	* Cutibacterium *	3	*Acnes*	0.47	1	3
Fungi	*Plasmopara*	4	*Obducens*	0.01	1	1
	*Cronartium*	3	*Quercuum*	0.05	1.17	2
	*Candida*	3	*Tropicalis*	0.09	1	3
	*Aspergillus*	5	*Rambellii*	0.05	1.51	3

A batch of 40 gr of bone cement containing 3 g of meropenem antibiotic non-articular pads was placed. A drainage tube was subsequently inserted after closing and injecting 200 mg of voriconazole into the joint cavity. The patient underwent a 3 week course of intravenous voriconazole and a 2 week course of voriconazole injections into the joint cavity every other day.

**Fig. 2.**
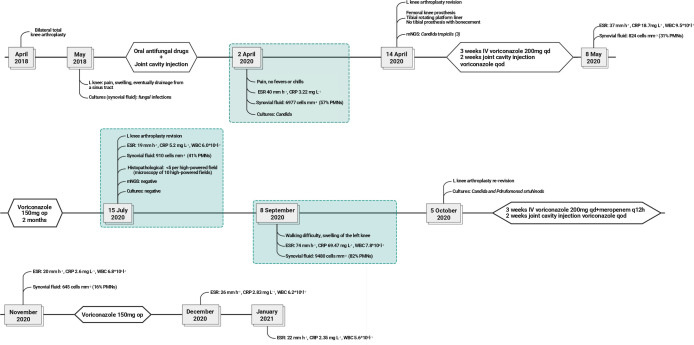
Timeline of clinical course with relevant laboratory findings listed. CRP, C-reactive protein (mg l^−1^); ESR, erythrocyte sedimentation rate (mm h^−1^); IV, intravenous; mNGS, metagenomic shotgun sequencing; PMNs, polymorphonuclear leukocytes; R, right; L, left.

At the end of the antifungal treatment, laboratory evaluation was substantial for erythrocyte sedimentation rate (ESR) of 37 mm h^−1^, CRP of 18.7 mg l^−1^ and white blood cell count of 9.5×10^9^ l^−1^, while 824 cellsmm^−3^ with 23 % neutrophils were revealed in synovial fluid analysis. ESR and CRP remained elevated, but the total number of polymorphonuclear cells, as well as the percentage of neutrophils in the joint fluid, were reduced. Hence, oral voriconazole 150 mg was administered once daily. After 2 months, ESR was 19 mm h^−1^, CRP was 5.2 mg l^−1^ and leucocyte count was 6×10^9^ l^−1^. Synovial fluid analysis showed 910 cells mm^−3^, of which 41 % were neutrophils. Under histopathological microscopy of 10 high-powered fields, fewer than five per high-powered field were intraoperative. A revision knee arthroplasty was consequently performed. Three tissue specimens were dispatched for aerobic and anaerobic bacterial and fungal cultures as well as mNGS testing; the culture results were negative. No disease-related pathogens were found during the mNGS testing. The micro-organisms detected by mNGS are presented in Table 2 and Fig. 2.

**Table 2.** The metagenomic shotgun sequencing report from 15 July 2020

Type	Genus	No. of reads	Species	Coverage rate (%)	Depth	No. of reads
Bacteria	* Burkholderia *	301	*Ambifaria*	13.3	1	3
			*Pyrrocinia*	3.08	1	6
			*Ubonensis*	3.45	1	8
			*Vietnamiensis*	7.9	1.01	122
	* Cupriavidus *	15	*Metallidurans*	1.46	1.03	10
	* Methylobacterium *	7	*Radiotolerans*	0.45	1	4
	* Cutibacterium *	5	*Acnes*	0.93	1	4
	* Methylorubrum *	5	*Extorquens*	0.22	1	4
Fungi	*Melampsora*	7	*Pinitorqua*	0.49	1.03	
	*Plasmopara*	3	*Halstedii*	0.00	1	0
	*Cronartium*	2	*Quercuum*	0.04	1.42	2
	*Aspergillus*	1	*Sydowii*	0.01	1	1
	*Clavaria*	1	*Fumosa*	0.02	1	1

Two months following the revision, the patient again experienced walking difficulties and swelling of the left knee with ESR of 74 mm h^−1^, CRP of 69.47 mg l^−1^ and white blood cell counts of 7.8×10^9^ l^−1^. The synovial fluid analysis showed 9480 cells mm^−3^ with 82 % neutrophils. After reimplantation, the patient was infected with fungal (*C. tropicalis*) and bacterial (* Pseudomonas aeruginosa *) infections, and thus underwent an intravenous course of voriconazole and meropenem after the procedure.

The patient was reassessed 3 months after revision surgery and reported that the knee was healing well, with no sign of infection with ESR of 22 mm h^−1^, CRP of 2.35 mg l^−1^ and a white blood cell count of 5.6×10^9^ l^−1^.

Fig. 2 displays the entire timeline of the patient’s diagnosis and treatment.

## Discussion

PJI is less often caused by fungal infections, but these infections, which are challenging to cure, may be growing in number [7]. Periprosthetic fungal infections have a death risk of up to 25 %, and patients with fungal infections have high failure rates after revision surgery in comparison to those with bacterial infections [8]. The most prevalent fungus is *Candida tropicalis* [3, 9]. Clinically, we are unable to distinguish between bacterial and fungal infections on the basis of the symptoms, inflammatory markers across the body and aspiration cell counts.

We currently rely on the findings of cultures from joint aspirations or tissue. *Candida* culture-based detection methods have a sensitivity of <50 % [10], with 51 % of their original cultures identified as potential contaminants [9, 11, 12]. Biofilm production is common in fungal infections, with *C. albicans* producing a more complex biofilm [13]. Fifty-five per cent of cases have been found to have bacterial co-infection [14]. This makes it more challenging to identify and categorize harmful micro-organisms. Moreover, culture typically takes 7 to 10 days [15]. Due to the lack of accessible, rapid and accurate diagnostic procedures, the price of antifungal medications has increased due to their widespread use. Therefore, a better method for making an early diagnosis is required, which is essential for determining the disease’s prognosis and preventing and managing PJI with *Candida*.

Metagenomic shotgun sequencing (mNGS), which has been evolving as a tool for clinical fungal identification, might provide a better solution [16]. In comparison to culture, mNGS diagnosis within 30 h can provide an early diagnosis [17]. The ability to detect universal pathogens irrespective of the type of microbe is another key advantage of mNGS [18]. It requires little or no prior knowledge of the pathogen [19]. Therefore, we suggest that mNGS be used to detect the genomic DNA of *C. tropicalis* in the periprosthetic tissue in order to optimize the use of anti-fungal agents for effective treatment.

In the present case, a total of 43 996 027 reads were obtained from periprosthetic tissue, after removing human genome reference sequence and low-complexity reads. The ultimate result was *C. tropicalis*, which was combined with the comparison of background micro-organisms and negative controls with quality control. The genomic sequence obtained for a given pathogen is dependent not only on the sequencing reads, coverage rate and depth, but likewise on the efficacy of human DNA removal, the bacterial load and the nature of the infection. A high degree of disease association must be taken into account. The higher the degree of disease association, the greater the likelihood of causing disease. On the other hand, not all micro-organisms determined by mNGS are associated with diseases, such as *Aspergillus rambellii* [20]. Since it is not related to the disease, it has occasionally been regarded as a background organism rather than a clinical pathogen and was not considered in this case. *Burkholderia vietnamiensis* and *Cutibacterium acnes* were also found in the current study. Although generally non-pathogenic, *C. acnes* in particular has been shown to cause joint infections in the literature [21–23]. Since disease is frequently multifactorial, we also believe that *C. tropicalis* may be important in our investigation based on the biomarker background library under quality control. Clinicians should therefore determine the actual causative agent(s) in accordance with mNGS results, the characteristics of the detected pathogen(s) and other examination results. Despite the fact that patients have co-infections (such as *Candida* and *Pseudomonas aeruginosa *) after reimplantation, mNGS technology has shown great superiority in microbiome detection, and more clinical data regarding the diagnosis criteria of infectious agents are needed in order to support the extensive usage of mNGS.

After the pathogen has been identified, the infection is treated by tissue debridement, with or without removal of the implant, and by long-term administration of anti-fungal agents. Given how devastating fungal infections can be, debridement alone is not adequate [9]. Antifungals, delayed reimplantation and implant removal have all led to positive results in a number of studies [24, 25]. Some studies indicate a positive impact of keeping a high concentration of fluconazole in joint fluid [26]. Despite the fact that patients who had delayed implantation had a 20 % chance of developing fungal infections again, numerous studies show that delayed reimplantation provides the best chance for a good functional outcome [24].

The patient in this case was reinfected following phase II revision. Due to bacterial co-infection, the patient was given fluconazole with meropenem in order to combat the infection. *C. albicans* produces a larger and more complex biofilm in comparison to other *Candida* species, therefore eradication with antifungals alone is difficult [9].

This case demonstrates a new PJI pathogen that is otherwise challenging to detect by conventional methods. mNGS has grown in importance as a test for PJI and is deemed to be a critical adjunct diagnostic tool. The utilizing pleural effusion as an mNGS test specimen is feasible in the diagnosis of PJI. Simultaneously, mNGS can help clinicians in providing new diagnostic ideas for pathogenic infections where a number of traditional diagnostic methods have failed and further assist in clinical decision-making.
